# Small Molecule Decoy of Amyloid-β Aggregation Blocks Activation of Microglia-Like Cells

**DOI:** 10.3233/JAD-231399

**Published:** 2024-09-24

**Authors:** Sho Oasa, Gefei Chen, Marianne Schultzberg, Lars Terenius

**Affiliations:** a Department of Clinical Neuroscience, Center for Molecular Medicine, Karolinska Institutet, Stockholm, Sweden; b Department of Biosciences and Nutrition, Karolinska Institutet, Huddinge, Sweden; c Department of Neurobiology, Care Sciences & Society, Division of Neurogeriatrics, Bioclinicum J10 : 30, Karolinska Institutet, Stockholm, Sweden

**Keywords:** Alzheimer’s disease, amyloid-β, cytokine, inflammation, interleukin, tumor necrosis factor

## Abstract

**Background:**

Aggregated forms of the amyloid-β (Aβ) peptides which form protofibrils and fibrils in the brain are signatures of Alzheimer’s disease (AD). Aggregates are also recognized by microglia, which in early phases may be protective and in later phases contribute to the pathology. We have identified several small molecules, decoys which interfere with Aβ oligomerization and induce other aggregation trajectories leading to aggregated macrostructures which are non-toxic.

**Objective:**

This study investigates whether the small-molecule decoys affect microglial activation in terms of cytokine secretion and phagocytosis of Aβ peptide.

**Methods:**

The effects of the decoys (NSC 69318, NSC 100873, NSC 16224) were analyzed in a model of human THP-1 monocytes differentiated to microglia-like cells. The cells were activated by Aβ_40_ and Aβ_42_ peptides, respectively, and after treatment with each decoy the secreted levels of pro-inflammatory cytokines and the Aβ phagocytosis were analyzed.

**Results:**

NSC16224, which generates a double-stranded aggregate of thin protofibrils, was found to block Aβ_40_- and Aβ_42_-induced increase in microglial secretion of pro-inflammatory cytokines. NSC 69318, selective for neurotoxicity of Aβ_42_, and NSC 100873 did not significantly reduce the microglial activation in terms of cytokine secretion. The uptake of Aβ_42_ was not affected by anyone of the decoys.

**Conclusions:**

Our findings open the possibility that the molecular decoys of Aβ aggregation may block microglial activation by Aβ_40_ and Aβ_42_ in addition to blocking neurotoxicity as shown previously.

## INTRODUCTION

Alzheimer’s disease (AD) is a neurodegenerative disease leading to dementia and severe disabilities for those afflicted. The neuropathological characteristics in the brain consist of so-called amyloid plaques, neurofibrillary tangles, neuronal cell death and inflammation. The amyloid plaques are aggregates of amyloid-β (Aβ) peptides, derived from the amyloid-β protein precursor (AβPP). Aβ is a potent pro-inflammatory signal inducing the activation of microglial cells and astrocytes, and the ensuing production and release of proinflammatory signals such as cytokines^1,2^ as well as cytotoxic factors.^3^ Proinflammatory cytokines have been shown to stimulate the synthesis of AβPP and its metabolism to Aβ peptides^2,4^ resulting in a vicious circle leading to continuous inflammation and nerve cell death.^5^ Inflammation is normally down-regulated when the injurious agent has been removed and this process has been named resolution of inflammation.^6^ The resolution is mediated by so called pro-resolving lipid mediators (SPMs)^7^ and a dysfunctional resolution leads to chronic inflammation, as seen, e.g., in diseases such as cancer, rheumatoid arthritis, and periodontal disease (see^8^). In AD, the continuous presence of Aβ peptides likely leads to a sustained inflammation. Furthermore, a dysfunction in the resolution in the AD brain is supported by reduced levels of SPMs in the brain^9–11^ and cerebrospinal fluid (CSF).^10,12^ Thus, together with a dysfunctional resolution, the presence of Aβ peptides would lead to a chronic inflammatory state that is harmful to the brain.

Aβ peptides readily form several different aggregates. After the first nucleation step, Aβ peptides form low-molecular weight oligomers (less than 8-mer) containing both fibrillar and non-fibrillar species.^13,14^ These further form protofibrils and mature fibrils or high-molecular weight oligomers. Among the different aggregational forms, oligomeric forms have been shown to be most toxic and prone to induce inflammation. To inhibit aggregation would be a way to reduce toxicity and inflammatory responses. In early work we identified a central sequence for aggregation, Aβ_16–20_ (KLVFF), and by adding this peptide (or an extended variant) we could block the aggregation.^15^ We searched the NCI compound depository (https://cactus.nci.nih.gov/download/nci/) for potential inhibitors of aggregation aiming at the first step in this process. The search identified a few compounds that strongly affected the aggregation trajectory which we named decoys. Aggregates were still formed, but they showed reduced toxicity, topology of aggregates was altered, and fibril formation was blocked.^16^ The remarkable topological changes might have consequences for neuroinflammation.

In the present study we investigated whether the identified compounds (decoys) also affected the pro-inflammatory effects of Aβ in a human microglia cell model. The main forms of Aβ identified in AD brain are Aβ_40_ and Aβ_42_, and the compounds were tested against both of these peptides.

## MATERIALS AND METHODS

### Small molecule compounds

Small molecule Aβ aggregation decoys have been described previously.^16^ In this paper we used the chemical compound names NSC 69318, NSC 100873, and NSC 16224 which were labelled as #5, #7, and #2-2 in the previous work. Information on the compounds is available *via* National Cancer Institute (NCI); Downloadable structure files of NCI Open Database Compounds: Release 4.2012. https://cactus.nci.nih.gov/download/nci/ (last accessed 17 September 2022). All compounds were dissolved in 20 mM HEPES buffer (pH 7.4) to 10 mM molar concentration as a stock solution. Molecular structures are shown in the Supplementary Figure 1.

### Aβ peptide preparation

The production and purification of Aβ peptides were performed as previously described.^17,18^ In brief, the 40 or 42 amino acid residues of Aβ, Aβ_1–40_ (Aβ_40_) or Aβ_1–42_ (Aβ_42_) were fused to the NT^*^ tag and expressed in BL21(DE3) *E. coli*. The fusion protein NT^*^-Aβ_40_ and NT^*^-Aβ_42_ were purified with a Ni-NTA column, cleaved by NT*-Tev^18^ and lyophilized. The lyophilized powder of each protein was solubilized in 20 mM Tris pH 8.0 with 7 M guanidium chloride, and the Aβ_40_ and Aβ_42_ monomers were isolated, respectively, by a Superdex30 26/600 column (Cytiva) in 1×phosphate buffered saline (1×PBS). The concentrations of Aβ_40_ and Aβ_42_ monomers were determined by measurement of absorbance at 280 nm and 300 nm with an extinction coefficient of 1424 M^–1^ cm^–1^ for (A280–A300). The purified Aβ_40_ and Aβ_42_ monomers were aliquoted in low-binding tubes and stored at –20°C. The oligomeric states of Aβ_42_ were analyzed using Thioflavin T (ThT) with confocal laser scanning microscopy (Supplementary Figure 2). ThT molecules were excited with 458 nm laser and fluorescence was detected in 530–610 nm.

Fluorescently labelled Aβ_42_, HiLyte Fluor^TM^ 488- Aβ_42_ (Aβ42–488) was purchased from AnaSpec, Fremont CA. The lyophilized powder of Aβ42–488 was reconstituted using the company protocol (https://www.anaspec.com/assets/d84c5199-d8fe-45b2-a1fb-3222206ad00b/tds-en-as-60479-01-beta-amyloid-1-42-hilyte-fluor-488-labeled.pdf). The 0.1 mg of Aβ42-488 was reconstituted by adding 50μL 1% NH_4_OH, followed by the dilution with 1×PBS as a 200μM stock solution. The stock solution was stored at –20°C.

### Cell culture

Human monocytic THP-1 cells (LGC Standards, Teddington, UK) were maintained at 37°C with 5% CO_2_ in RPMI1640 modified with L-glutamine, HEPES, Phenol Red, sodium pyruvate, high glucose and low sodium bicarbonate (A1049101; Gibco) and supplemented with 10% fetal bovine serum (FBS) and 50 nM β-mercaptoethanol. THP-1 cells were spun down at 200×g for 5 min. Cell pellets were suspended with culture medium described above, seeded in T175 red-capped cell culture flasks (Sarstedt, Helsingborg, Sweden).

For THP-1 cell differentiation, the pellet of THP-1 cells after the spin-down was suspended with RPMI1640 medium containing 50 ng/mL phorbol 12-myristate 13-acetate (PMA) according to previously used protocol.^19^ THP-1 cells (2.5×10^4^ cells/well or 6.0×10^5^ cells/well) were seeded in 24-well plates (VWR) for ELISA or 6-well plates (VWR) for phagocytosis analysis. The cells were differentiated for 3 days.

### ELISA for secreted IL-6 and TNF-*α* levels

Stock solutions of 179μM Aβ_40_ and 40μM Aβ_42_ in 1×PBS were slowly thawed on ice to avoid aggregation and diluted to 10μM Aβ_40_ and 5μM Aβ_42_ with RPMI1640 medium in low-binding tubes. The same molar concentration of the small molecule compounds was added to the Aβ solution (10μM in Aβ_40_ and 5μM in Aβ_42_ solution, respectively). The differentiated THP-1 (dTHP-1) cells were incubated with 350μL of these combined solutions, with Aβ_40_ or Aβ_42_ (each alone), or with the compounds alone in RPMI1640 medium as negative control at 37°C for 24 h after which the medium was collected in low-protein binding tubes and stored shortly at–20°C.

ELISA experiments were performed according to the protocol for human interleukin-6 (IL-6) DuoSet ELISA and human tumor necrosis factor (TNF)-*α* DuoSet ELISA (DY206-05 and DY210-05; Biotechne R& D systems). Briefly, capture antibodies were absorbed in 96-well black plates (Thermo Fisher) and excess antibodies were washed after 24 h with PBS containing 0.05% Tween-20. The plates were incubated with blocking buffer (PBS with 1% BSA). The samples of culture media were thawed and centrifuged at 1000×g for 5 min, and the supernatant collected and diluted twice with the blocking buffer, followed by adding 100μL of the mixture to the plates. On the same plates, recombinant human IL-6 or recombinant human TNF-*α* were added in different dilutions for the standard curves according to the instructions by the company. Following incubation with detection antibodies and Streptavidin-HRP, the plates were incubated with HRP substrate in QuantaRed Enhanced Chemifluorescent HRP Substrate Kit (Thermo Fisher) for 10 min, and the signals were recorded by a plate reader, CLARIOstar*^*Plus*^* (BMG LABTECH).

Data analysis was performed using two softwares, CLARIOstar – Data Analysis and SMART Control Data Analysis. Fitting a four-parameter logistic curve to standard data from both recombinant IL-6 and TNF-*α* (Supplementary Figure 3), the fluorescence signal in each sample was transformed to the concentration of IL-6 and TNF-*α* respectively.

### Phagocytosis analysis using flow-cytometry

Stock solution of 200μM Aβ42–488 was diluted to 200 nM molar concentration with RPMI1640. The same molar concentration of the compounds was added to the Aβ42–488 solution as sample or to RPMI1640 as negative control. dTHP-1 cells seeded in 6-well plates were incubated at 37°C for 20 min with 1 mL of the following solutions: each small molecule compound alone, Aβ42–488 alone or both Aβ42–488 and compounds. Subsequently, the dTHP-1 cells were washed twice with PBS and incubated with 2 mL TryPLE Select (Gibco) at 37°C for 20 min. Bright-field microscopy confirmed detachment of the cells from the plates. The cell-containing solution was centrifuged at 1530 g for 10 min and the resulting pellet washed twice with FACS buffer (PBS with 2% FBS). After the final centrifugation, the cells were fixed with 4% paraformaldehyde in PBS at room temperature for 45 min, followed by washing twice with FACS buffer. Finally, the dTHP-1 cells were suspended in PBS and 50,000 cells were loaded in a flow-cytometer (BD Accuri™ C6 Plus Flow Cytometer) for analysis of phagocytosis of Aβ42–488. Untreated dTHP-1 cells were analyzed in the flow-cytometer to set the region of interest (ROI) for data collection by scattering light.

To calculate phagocytic cell numbers and average fluorescence intensity, a threshold of autofluorescence intensity was set as the fluorescence intensity with 1% cross-section of untreated dTHP-1 cells.

### Confocal laser scanning microscopy (CLSM) imaging of Aβ_42_ phagocytosis

The THP-1 cells (2.0×10^4^ cells/well) were differentiated on the 96-well chambered cover glass (μ-Plate 96 well Black; ibidi) for 3 days. After differentiation, the cells were incubated with 200 nM Aβ42–488 in the absence or presence of the same molar concentration of small molecule decoys (NSC 69318, NSC 100873, NSC 16224) for 20 min. The cells were washed twice with phenol-red free RPMI 1640 medium (Gibco). The cells were further stained with Hoechst33342 (NucBlue Live ReadyProbes Reagent; Invitrogen) followed by washing twice with phenol-red free RPMI 1640 medium.

CLSM imaging was performed using the LSM880 (Carl Zeiss) microscope system equipped with a 405 nm laser, 488 nm Ar-ion laser, and a water immersion objective lens (C-Apochromat, 40×, 1.2 N.A., Corr, Carl Zeiss), a gallium arsenide phosphide (GaAsP) detector and photomultiplier tube (PMT) detectors. Hoechst33342 and HiLyte488 were excited using the 405 nm laser and 488 nm laser, respectively. The pinhole size was adjusted to 1 AU (32μm in Hoechest33342 ch.; 39μm in HiLyte488 ch.). Both fluorescence signals were split by diffraction grating, introduced to a PMT detector (Hoechst33342; 410–510 nm) and a GaAsP detector (HiLyte488; 493–630 nm). To avoid crosstalk signal, the multi-track mode was used. During imaging with the laser at 488 nm, another PMT detector collected the transmitted laser to confirm intact cell morphology.

### Statistics

All statistical analyses were performed using the Origin Data Analysis and Graphing Software (OriginLab Corporation, USA). The results are expressed as scatter plots in bar graphs as mean±SD. *p* < 0.05 was considered statistically significant. One-way ANOVA were used to test for group differences, with the Tukey *post hoc* test. Results of all combinations in Tukey *post hoc* test were shown in Supplementary Tables 1–3.

## RESULTS

### Small-molecule decoy reduced the pro-inflammatory effects of Aβ peptides

**Fig. 1:**
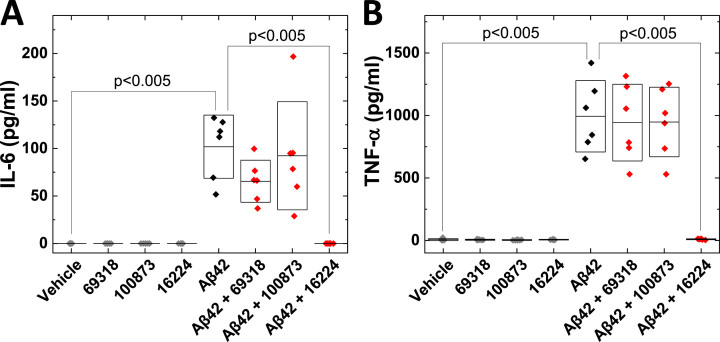
**Compound NSC 16224 inhibits Aβ_**42**_-induced secretion of IL-6 and TNF-*α* from human microglia-like cells.** Differentiated human THP-1 (dTHP-1) cells were incubated with vehicle, 5μM of each compound, 5μM Aβ_42_, or 5μM Aβ_42_ _ +_ 5μM of the compound for 24 h. IL-6 (A) and TNF-*α* (B) were analyzed by ELISA in 6 independent experiments.

We have previously characterized the effects of small-molecule decoys on Aβ aggregation and cytotoxicity.^16^ Since Aβ is known to induce pro-inflammatory effects, including secretion of the pro-inflammatory cytokines IL-6 and TNF-*α* from human microglia,^19^ we assessed the effect of the small molecule decoys on the secretion of IL-6 and TNF-*α* (Fig. 1). The dTHP-1 cells were incubated with Aβ_42_ and/or small-molecule decoys (NSC 69318, NSC 100873, NSC 16224). The decoy molecules alone did not affect the secretion of IL-6 or TNF-*α*, whereas presence of Aβ_42_ induced their secretion from the dTHP-1 cells (Fig. 1).

NSC 16224 was found to block the Aβ_42_-induced increase in released cytokines. Earlier studies showed that oligomeric Aβ_42_ was more potent in inducing microglial TNF-*α* production than fibrillar Aβ_42_ ^20^ suggesting that NSC 16224 suppressed formation of oligomeric forms of Aβ_42_. NSC 100873 which was characterized as an inducer of sheet-like Aβ aggregates^16^ had no effect (Fig. 1). NSC 69318 which was found to selectively affect Aβ_42_ aggregation^16^ slightly reduced IL-6, although not reaching statistical significance, and had no effect on the TNF-*α* levels (Fig. 1).

**Fig. 2:**
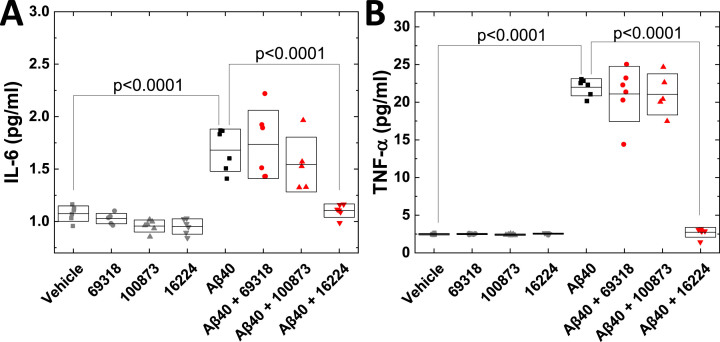
**Compound NSC 16224 inhibits Aβ_**40**_-induced secretion of IL-6 and TNF-*α* from human microglia-like cells.** Differentiated human THP-1 (dTHP-1) cells were treated with vehicle, 10μM of each compound, 10μM Aβ_40_, or 10μM Aβ_40_ + 10μM of the compound alone for 24 h. IL-6 (A) and TNF-*α* (B) were analyzed by ELISA. Six (6) independent experiments were performed.

Aβ_40_ is also a major form of Aβ found in AD brains, and we therefore further characterized the effects of small-molecule decoys on the pro-inflammatory responses induced by Aβ_40_ (Fig. 2). Aβ_40_ induced the secretion of both IL-6 and TNF-*α*, but the induction was 50–100 times less than that by Aβ_42_ even when using the double concentration of Aβ_40_, suggesting that the microglia-like cells are more responsive to Aβ_42_ oligomers than to those of Aβ_40_. NSC 16224 blocked the increase in IL-6 and TNF-*α* secreted from the cells induced by Aβ_40_, similarly to Aβ_42_. NSC 100873 had no effect as shown for Aβ_42_. NSC 69318 showed no effect on IL-6, suggesting that this compound may have a selective effect on signaling pathways activated by Aβ_42_ oligomers as previously found in neurotoxicity.^16^

### Small-molecule decoys had no statistically significant effect on phagocytosis of Aβ

**Fig. 3:**
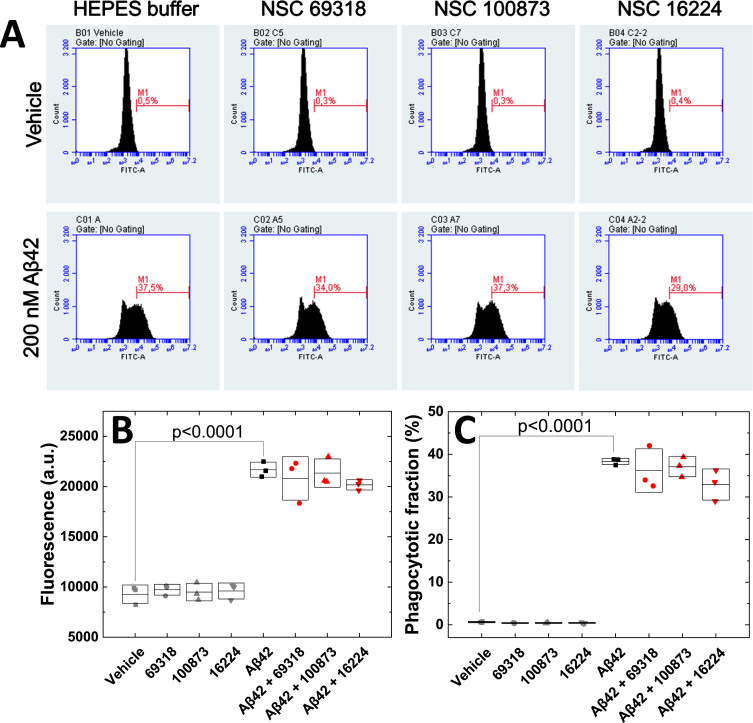
**Analysis of Aβ_**42**_ phagocytosis by human microglia-like cells.** Phagocytosis of Aβ_42_ by differentiated human THP-1 (dTHP-1) cells was analyzed using flow-cytometry. Differentiated dTHP-1 cells were incubated with vehicle, 200 nM Aβ42-488, 200 nM of each compound, or 200 nM Aβ42-488 + the 200 nM compound. A) Distribution of fluorescence intensity of Aβ42-488 in the dTHP-1 cells. Average fluorescence intensity (B) and percentage of dTHP-1 cells that had taken up Aβ42-488 (C). Three independent experiments were performed.

Microglia have been shown to take up Aβ_42_ by phagocytosis.^20^ We assessed the effects of the small molecule decoys on the phagocytosis of fluorescently labeled Aβ_42_ (Aβ42–488). Confocal Laser Scanning Microscopy (CLSM) confirmed the phagocytosis of Aβ42–488 into live dTHP-1 cells also under the co-treatment with small molecule decoys (Supplementary Figure 4). To analyze the fluorescence intensity in each single cell we used flow cytometry. The distribution of fluorescence intensity showed no difference between treatment with decoys or vehicle, and the Aβ42-488-treated dTHP-1 cells showed higher fluorescence intensity level than decoy treatment alone (Fig. 3A). NSC 69318 and NSC 100873 had no effect on phagocytosis of Aβ42-488 by the dTHP-1 cells. NSC 16224 slightly reduced the average fluorescence intensity and the fraction of dTHP-1 cells that had taken up Aβ42–488, although not reaching statistical significance (Fig. 3). Fibrillar Aβ_42_ is more prone to be taken up by microglia cells,^20^ indicating that NSC 16224 may change the direction of aggregation from oligomeric to a non-toxic form which is not phagocytosed.

## DISCUSSION

The aggregation of Aβ is associated with AD. Aggregation can be initiated by seeding and is dependent on time and concentration. Under laboratory conditions it is convenient to use higher concentrations to avoid lag phase and variable kinetics.^21,22^ The significance of this process receives indirect support from clinical studies of anti-amyloid antibodies: aducanumab,^23^ now discontinued, lecanemab^24^ and donanemab.^25^ Lecanemab (Leqembi^®^) is now approved by the U.S. Food and Drug Administration (FDA) for mild dementia of AD.

This study was initiated by our observation that selected small molecules named decoys interfere with Aβ aggregation toxicity.^16^ These small molecules were selected to interact already at the dimerization/oligomerization stages. Several of these molecules were very potent, suggesting specific interactions. Unexpectedly, the induced aggregation pathways differed dramatically resulting in no formation of fibrils, but formation of protofibrils, with buds and branches never observed in controls, a 2D-network and a double-stranded thin protofibril. Since neuroinflammation is a characteristic of AD^26^ and may even precede plaque formation, we decided to investigate whether the small molecules would interfere with the activation of microglia. Activated microglia produce and secrete inflammatory factors including cytokines^1,2^ and cytotoxic factors,^3^ providing an environment that when sustained for long times, such as due to continuous presence of Aβ, leads to loss of homeostasis and neuronal cell death. In the acute situation, however, the activities of microglia are beneficial by sensing and removing the injurious agent to restore homeostasis (see^27^). The findings from studies on the effects of anti-amyloid treatment in AD mouse models^28^ further emphasizes the important role of microglia in relation to amyloid and to further characterize their responses. Transcriptomic profiling studies of microglia in health and disease^29–32^ add to our knowledge of the complexity and subpopulations of microglia and indicate the importance of careful characterization of cellular populations to interpret data.

Although only differing by two amino acids, Aβ_40_ and Aβ_42_ are distinctly different with respect to the aggregation pathway and role in neuropathology.^33^ Already at the dimer stage cryo-EM shows that the Aβ_42_ interface is longer and kinked whereas the Aβ_40_ interface is flat.^34^ Whereas Aβ_40_ is typically detected in cerebral amyloid angiopathy,^35^ there is general agreement that Aβ_42_ is the species relevant for neurotoxicity in AD.

In our experiments, Aβ_42_ aggregation strongly activated the microglia-like cells, whereas the Aβ_40_ variant was considerably less potent and had lower efficacy. Interestingly, NSC 16224 completely blocked the activation by both peptides. NSC 69318 was weakly active, but only with Aβ_42_ in line with the previously shown selective activity against Aβ_42_-induced toxicity.^16^ NSC 100873 was not active.

There are several receptors that have been shown to bind Aβ aggregates such as β1 integrin, formyl peptide receptor-like 1 (FPRL1) and receptor for advanced glycosylation end-products (RAGE), which all bind both monomeric and fibril Aβ.^36^ The scavenger receptors A and BI bind only monomeric Aβ.^36^ The shuttling-protein nucleolin was shown to be a receptor for Aβ on microglial cells.^37^ Interestingly, nucleolin strongly associated with Aβ_42_ and phagocytosed both the monomeric and fibril forms, whereas Aβ_40_ was only taken up weakly.^37^ The results also indicated that binding of nucleolin to Aβ_42_ was neither ionic nor hydrophobic and that it was dependent on the structure of Aβ_42_ contributing to its aggregation. In a recent study on rat glial cells, RNA sequencing data showed clear differences in the response to Aβ_40_ and Aβ_42_ fibrils.^38^ This was evident in primary cultures of both astrocytes and microglia, with about 10× more genes upregulated by Aβ_42_ than by Aβ_40_ in rat microglia, and pathway analysis showed that Aβ_42_ treatment resulted in induction of immune response pathways.^38^ The data also showed that genes found in so called disease-associated microglia (DAM) were markedly upregulated by Aβ_42_, whereas most of these genes were down-regulated by Aβ_40_, notably including *Trem2*, a key DAM-triggering receptor gene.^38^ In postmortem brains from patients with Down syndrome^39^ and AD,^40^ microglial cells were more often found in association with amyloid plaques containing both Aβ_40_ and Aβ_42_ compared to those containing only Aβ_42_.

The interaction between amyloid and brain immunity is dynamic with a protective effect in prodromal phases of the disease. As the disease progresses, activated microglia due to continuous presence of Aβ, may have the opposite effect, releasing cytokines and other mediators which exert a neurotoxic effect.^41^ This sequence of events could be blocked by one of our decoys, NSC 16224, and regarding Aβ_42_ aggregation reduced by NSC 69318. This means that we would have a tool that can address two aspects of the disease process in the Alzheimer brain, neuroinflammation and neurotoxicity.^16^

Already in our previous publication,^16^ we discussed toxicology as a function of aggregate architecture recorded by TEM analysis. NSC 16224 stands out in generating a double-stranded thin filament aggregate which was shown to block both cytotoxicity^16^ and now microglial activation. This profile would seem highly interesting for exploitation therapeutically. We have argued that the effect of NSC 69318 is related to its interaction with the Aβ COOH-terminus, which has been found in other studies to be very hydrophobic and significant for aggregation.^42^ An exotic induction of a 2-dimensional network with NSC 100873 adds further suggestions that altered surface morphology of aggregation may lessen neurotoxicity but not necessarily microglialactivation.

It may also be significant that aggregate surface molecular patterns have a direct influence on microglia reactivity. The recent developments of neuroimplants have stimulated work on surface properties. It has been shown that a fibrous surface matrix is more challenging than a smooth area^43^ and that lipid droplets are accumulated in microglia associated with plaques.^44^ In analogy, the unique smooth double-stranded fibril observed with NSC 16224 may not be recognized by the microglial radar.

Aggregation mechanisms and functionality are an area of nanobiological interest.^45^ As already observed,^16^ Aβ has several epitopes for binding leading to aggregation. Inhibition of aggregation is therefore challenging and changing aggregation trajectory may be a mechanism for further study and eventually therapy.

### Conclusions

In conclusion, the molecular decoys of Aβ open the possibility to block neurotoxicity and microglia activation of Aβ_40_ and Aβ_42_ by NSC 16224, or to block only Aβ_42_ neurotoxicity and reduce microglia activation by NSC 69318. Selectivity of action is a key in drug development. Since Aβ_42_ is more toxic, selective interaction with this peptide may be significant for further analyses. NSC 100873 was only found to block neurotoxicity but not microglial activation, another possible link to selective action. As a principle, early intervention in pathogenesis should be preferable.

### Limitations to the study

Studies on human neuroinflammation at a mechanistic level require relevant and methodologically practical *in vitro* models. Human primary microglia are difficult to obtain, and other alternatives need to be considered. The present work was performed in a model of PMA-differentiated human THP-1 (dTHP-1) cells, a model widely used in studies on macrophages and microglia.^46^ The differentiation converts the THP-1 cells, a leukemia monocytic cell line, to macrophages. Macrophages and microglia share many properties and many studies have indicated that THP-1 cells and microglia respond to Aβ in a similar pattern, and therefore Aβ-treated THP-1 cells are frequently used to study AD-like neuroinflammation.^47–51^ An alternative to the dTHP-1 cells is to use peripheral monocytes from AD patients differentiated to macrophages, a model that has been used successfully rendering important findings.^52^ Although this is a more relevant model compared to the THP-1 cells, the advantage of using a cell line is the unlimited supply. For future studies a further alternative is to use human pluripotent stem cells from AD patients and healthy controls for higher physiological relevance and sufficient supply for analyses requiring large materials.

The concentrations of Aβ_40_ and Aβ_42_ used in the present study are considerably higher than those measured in human CSF,^53^ but commonly used for *in vitro* studies.^54^ Furthermore, local concentrations of Aβ peptides in the AD brain are probably higher than levels in the CSF,^55^ and reactive microglia are co-localized with amyloid plaques.^56^

Promising effects obtained in *in vitro* studies may, however, meet with challenges to translate to *in vivo* applications. One challenge is passage through the blood-brain barrier (BBB) since NSC 16224 (830 Da) is larger than the restriction barrier of molecular weight (<400 Da) for a passive diffusion across the BBB.^57^ Linking the compound to a transporter may be helpful for passage through the BBB. Also, intranasal administration may bypass the barrier.

## AUTHOR CONTRIBUTIONS

Sho Oasa (Conceptualization; Data curation; Formal analysis; Methodology; Writing – original draft; Writing – review & editing); Gefei Chen (Methodology; Resources; Writing – review & editing); Marianne Schultzberg (Conceptualization; Funding acquisition; Methodology; Resources; Supervision; Writing – original draft; Writing – review & editing); Lars Terenius (Conceptualization; Funding acquisition; Investigation; Supervision; Writing – original draft; Writing – review & editing).

## Supplementary Material

Supplementary Material

## Data Availability

The data supporting the findings of this study are available within the article and/or its supplementary material.
