# Supplementary material for: Small Molecule Decoy of Amyloid-β Aggregation Blocks Activation of Microglia-Like Cells
Source: J Alzheimers Dis. 2024 Sep 24;101(3):787–96. doi: 10.3233/JAD-231399 (PMC11492064; doi:10.3233/JAD-231399)
Supplement: Supplementary Material [file jad-101-jad231399-s001.pdf]

Supplementary Material

Small Molecule Decoy of Amyloid-β Aggregation Blocks Activation of Microglia-Like Cells

Compound name	Structure
NSC 69318	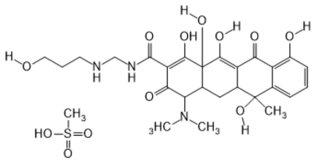
NSC 100873	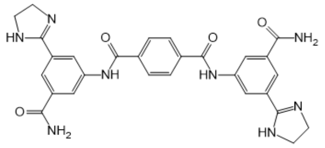
NSC 16224	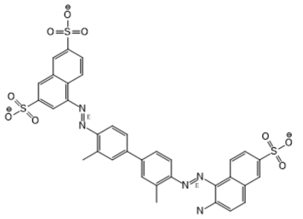

Supplementary Figure 1. Small molecule decoy structures.

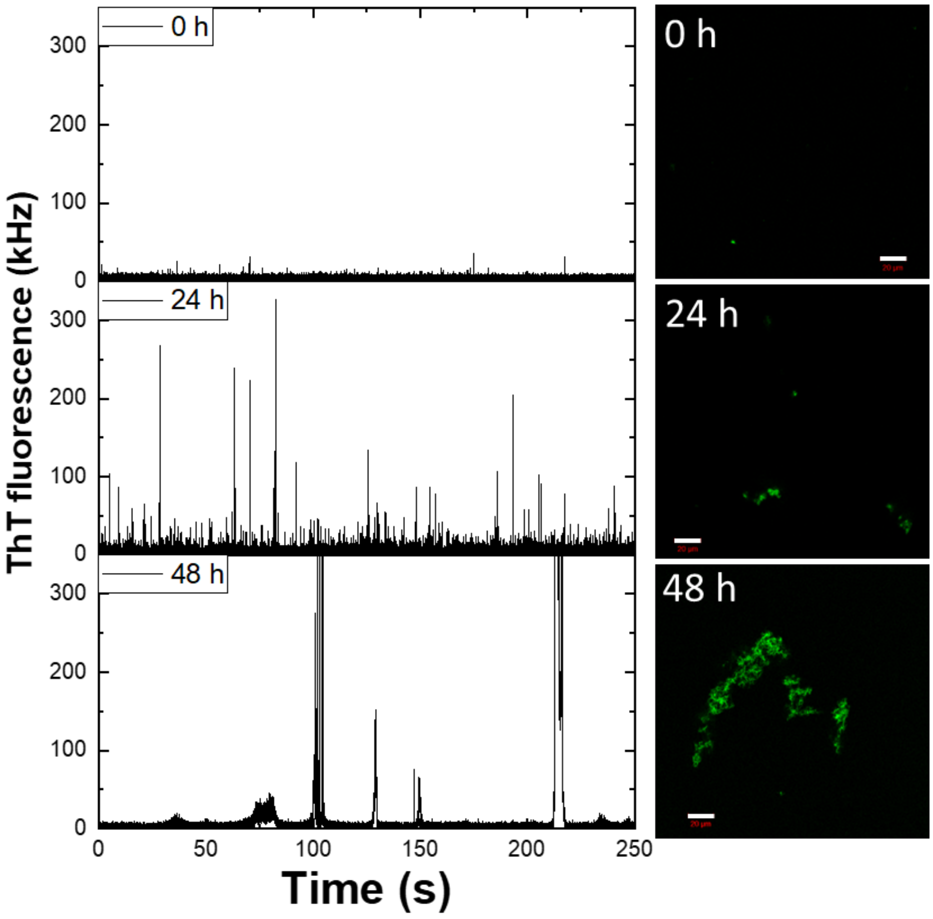

Supplementary Figure 2. Recombinant amyloid-β 42 (Aβ$_{42}$) aggregation state in treatment medium. Recombinant Aβ$_{42}$ peptide in PBS was diluted with phenol-red free RPMI medium to 10 µM concentration and mixed with 20 µM Thioflavin T (ThT). Fluorescence signals over time were recorded in the solution in the confocal microscope without scanning. Each fluorescent burst corresponds to the event passing ThT-positive oligomers through the confocal observation area (Left). Fluorescence images are taken of the cover glass to show the precipitated ThT-positive large aggregates. Scale bar: 20 µm (Right).

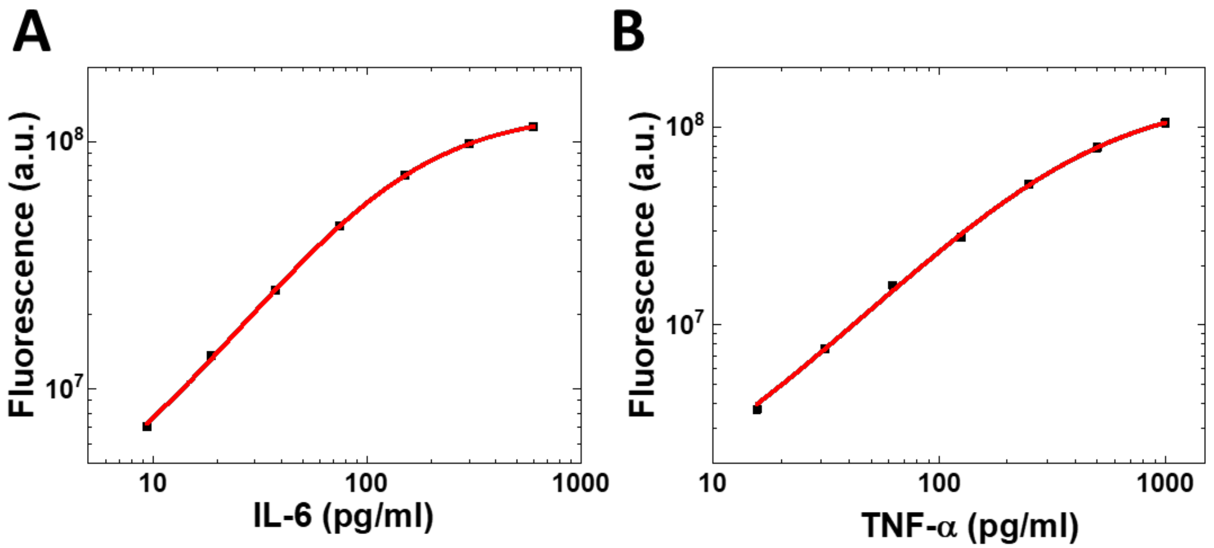

Supplementary Figure 3A-B. Standard curve in ELISA. Standard curves were generated for interleukin 6 (IL-6) (A) and tumor necrosis factor (TNF)-α (B). Black: experimental data points, Red: four-parameter logistic curve fitting. A.U., arbitrary units

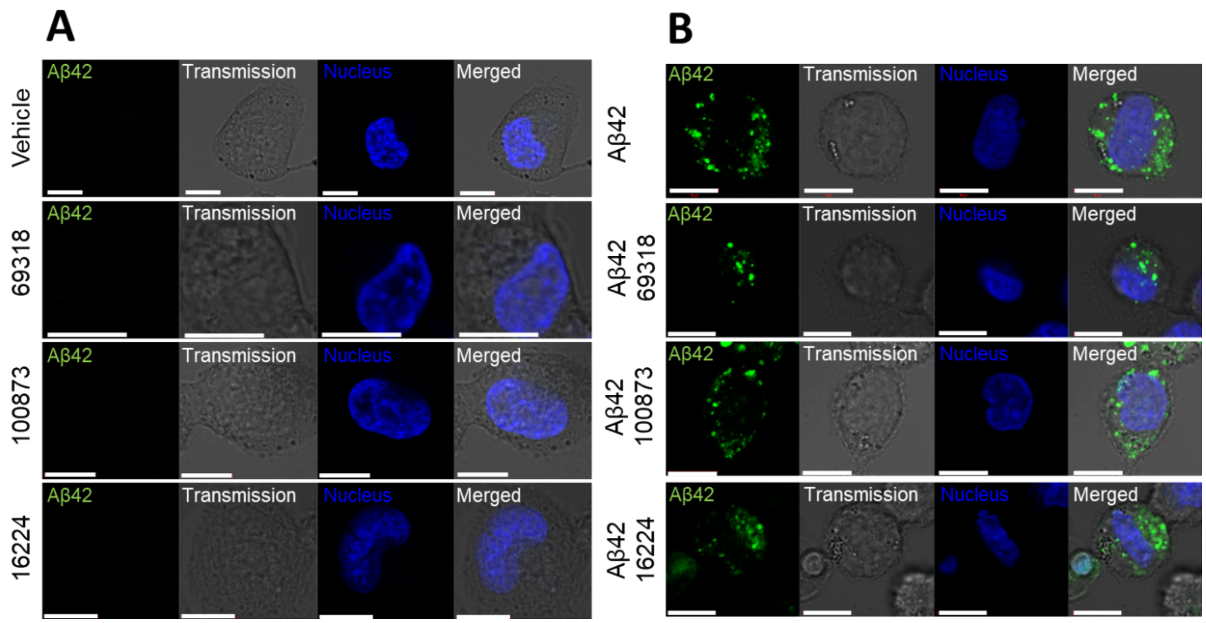

Supplementary Figure 4A-B. Aβ$_{42}$ phagocytosis in live dTHP-1 cells. Confocal Laser Scanning Microscopy (CLSM) imaging of live dTHP-1 cells treated with vehicle and small-molecule decoys (NSC 69318, NSC 100873 and NSC 16224) as negative controls (A) or incubated with 200 nM fluorescently labelled Aβ42 and the same molar concentration of decoys (B). Scale bars: 10 µm.

A

Data 1	Data 2	MeanDiff	Prob	Alpha
Vehicle	NSC 69318	0	1	0.05
Vehicle	NSC 100873	0	1	0.05
Vehicle	NSC 16224	0	1	0.05
Vehicle	Aβ42	101.87592	4.29E-07	0.05
Vehicle	Aβ42 + NSC 69318	65.4925	9.76E-04	0.05
Vehicle	Aβ42 + NSC 100873	92.36025	2.60E-06	0.05
Vehicle	Aβ42 + NSC 16224	0	1	0.05
NSC 69318	NSC 100873	0	1	0.05
NSC 69318	NSC 16224	0	1	0.05
NSC 69318	Aβ42	101.87592	4.29E-07	0.05
NSC 69318	Aβ42 + NSC 69318	65.4925	9.76E-04	0.05
NSC 69318	Aβ42 + NSC 100873	92.36025	2.60E-06	0.05
NSC 69318	Aβ42 + NSC 16224	0	1	0.05
NSC 100873	NSC 16224	0	1	0.05
NSC 100873	Aβ42	101.87592	4.29E-07	0.05
NSC 100873	Aβ42 + NSC 69318	65.4925	9.76E-04	0.05
NSC 100873	Aβ42 + NSC 100873	92.36025	2.60E-06	0.05
NSC 100873	Aβ42 + NSC 16224	0	1	0.05
NSC 16224	Aβ42	101.87592	4.29E-07	0.05
NSC 16224	Aβ42 + NSC 69318	65.4925	9.76E-04	0.05
NSC 16224	Aβ42 + NSC 100873	92.36025	2.60E-06	0.05
NSC 16224	Aβ42 + NSC 16224	0	1	0.05
Aβ42	Aβ42 + NSC 69318	-36.38342	0.20043	0.05
Aβ42	Aβ42 + NSC 100873	-9.51567	0.99736	0.05
Aβ42	Aβ42 + NSC 16224	-101.87592	4.29E-07	0.05
Aβ42 + NSC 69318	Aβ42 + NSC 100873	26.86775	0.56452	0.05
Aβ42 + NSC 69318	Aβ42 + NSC 16224	-65.4925	9.76E-04	0.05
Aβ42 + NSC 100873	Aβ42 + NSC 16224	-92.36025	2.60E-06	0.05

B

Data 1	Data 2	MeanDiff	Prob	Alpha
Vehicle	NSC 69318	-1.46152	1	0.05
Vehicle	NSC 100873	-3.53079	1	0.05
Vehicle	NSC 16224	-0.54165	1	0.05
Vehicle	Aβ42	986.58181	1.81E-08	0.05
Vehicle	Aβ42 + NSC 69318	935.89848	4.69E-08	0.05
Vehicle	Aβ42 + NSC 100873	940.76014	4.64E-08	0.05
Vehicle	Aβ42 + NSC 16224	2.73864	1	0.05
NSC 69318	NSC 100873	-2.06927	1	0.05
NSC 69318	NSC 16224	0.91987	1	0.05
NSC 69318	Aβ42	988.04333	1.80E-08	0.05
NSC 69318	Aβ42 + NSC 69318	937.36	4.68E-08	0.05
NSC 69318	Aβ42 + NSC 100873	942.22167	4.62E-08	0.05
NSC 69318	Aβ42 + NSC 16224	4.20016	1	0.05
NSC 100873	NSC 16224	2.98914	1	0.05
NSC 100873	Aβ42	990.1126	1.79E-08	0.05
NSC 100873	Aβ42 + NSC 69318	939.42927	4.65E-08	0.05
NSC 100873	Aβ42 + NSC 100873	944.29093	4.60E-08	0.05
NSC 100873	Aβ42 + NSC 16224	6.26943	1	0.05
NSC 16224	Aβ42	987.12346	1.80E-08	0.05
NSC 16224	Aβ42 + NSC 69318	936.44012	4.69E-08	0.05
NSC 16224	Aβ42 + NSC 100873	941.30179	4.63E-08	0.05
NSC 16224	Aβ42 + NSC 16224	3.28028	1	0.05
Aβ42	Aβ42 + NSC 69318	-50.68333	0.99963	0.05
Aβ42	Aβ42 + NSC 100873	-45.82167	0.99981	0.05
Aβ42	Aβ42 + NSC 16224	-983.84318	1.82E-08	0.05
Aβ42 + NSC 69318	Aβ42 + NSC 100873	4.86167	1	0.05
Aβ42 + NSC 69318	Aβ42 + NSC 16224	-933.15984	4.73E-08	0.05
Aβ42 + NSC 100873	Aβ42 + NSC 16224	-938.02151	4.67E-08	0.05

Supplementary Table 1A-B. Analysis of interleukin-6 (IL-6) (A) and tumor necrosis factor (TNF)-α (B) by ELISA after incubation of dTHP-1 cells with $A\beta_{42}$ and the small molecule compounds as shown in Fig. 1. MeanDiff: mean difference between data 1 and data 2, Prob: probability value for significant difference, Alpha: criterion of significance. Significant differences are highlighted in yellow.

A

Data 1	Data 2	MeanDiff	Prob	Alpha
Vehicle	NSC 69318	-0.04767	0.99965	0.05
Vehicle	NSC 100873	-0.11825	0.92275	0.05
Vehicle	NSC 16224	-0.12292	0.90705	0.05
Vehicle	Aβ40	0.60483	6.97E-06	0.05
Vehicle	Aβ40 + NSC 69318	0.65917	1.37E-06	0.05
Vehicle	Aβ40 + NSC 100873	0.46747	0.00112	0.05
Vehicle	Aβ40 + NSC 16224	0.02792	0.99999	0.05
NSC 69318	NSC 100873	-0.07058	0.99568	0.05
NSC 69318	NSC 16224	-0.07525	0.99362	0.05
NSC 69318	Aβ40	0.6525	1.66E-06	0.05
NSC 69318	Aβ40 + NSC 69318	0.70683	2.90E-07	0.05
NSC 69318	Aβ40 + NSC 100873	0.51513	2.71E-04	0.05
NSC 69318	Aβ40 + NSC 16224	0.07558	0.99344	0.05
NSC 100873	NSC 16224	-0.00467	1	0.05
NSC 100873	Aβ40	0.72308	1.86E-07	0.05
NSC 100873	Aβ40 + NSC 69318	0.77742	5.68E-08	0.05
NSC 100873	Aβ40 + NSC 100873	0.58572	3.12E-05	0.05
NSC 100873	Aβ40 + NSC 16224	0.14617	0.80225	0.05
NSC 16224	Aβ40	0.72775	1.64E-07	0.05
NSC 16224	Aβ40 + NSC 69318	0.78208	5.27E-08	0.05
NSC 16224	Aβ40 + NSC 100873	0.59038	2.71E-05	0.05
NSC 16224	Aβ40 + NSC 100873	0.15083	0.77641	0.05
Aβ40	Aβ40 + NSC 69318	0.05433	0.99918	0.05
Aβ40	Aβ40 + NSC 100873	-0.13737	0.87562	0.05
Aβ40	Aβ40 + NSC 16224	-0.57692	1.72E-05	0.05
Aβ40 + NSC 69318	Aβ40 + NSC 100873	-0.1917	0.57406	0.05
Aβ40 + NSC 69318	Aβ40 + NSC 16224	-0.63125	2.99E-06	0.05
Aβ40 + NSC 100873	Aβ40 + NSC 16224	-0.43955	0.00253	0.05

B

Data 1	Data 2	MeanDiff	Prob	Alpha
Vehicle	NSC 69318	0.02692	1	0.05
Vehicle	NSC 100873	-0.06142	1	0.05
Vehicle	NSC 16224	0.07333	1	0.05
Vehicle	Aβ40	19.51483	3.85E-08	0.05
Vehicle	Aβ40 + NSC 69318	18.62058	5.37E-07	0.05
Vehicle	Aβ40 + NSC 100873	18.56985	1.60E-07	0.05
Vehicle	Aβ40 + NSC 16224	0.25875	0.99999	0.05
NSC 69318	NSC 100873	-0.08833	1	0.05
NSC 69318	NSC 16224	0.04642	1	0.05
NSC 69318	Aβ40	19.48792	2.78E-06	0.05
NSC 69318	Aβ40 + NSC 69318	18.59367	5.36E-07	0.05
NSC 69318	Aβ40 + NSC 100873	18.54293	1.59E-07	0.05
NSC 69318	Aβ40 + NSC 16224	0.23183	1	0.05
NSC 100873	NSC 16224	0.13475	1	0.05
NSC 100873	Aβ40	19.57625	3.85E-08	0.05
NSC 100873	Aβ40 + NSC 69318	18.682	5.40E-07	0.05
NSC 100873	Aβ40 + NSC 100873	18.63127	1.60E-07	0.05
NSC 100873	Aβ40 + NSC 16224	0.32017	0.99997	0.05
NSC 16224	Aβ40	19.4415	2.77E-06	0.05
NSC 16224	Aβ40 + NSC 69318	18.54725	5.34E-07	0.05
NSC 16224	Aβ40 + NSC 100873	18.49652	1.59E-07	0.05
NSC 16224	Aβ40 + NSC 100873	0.18542	1	0.05
Aβ40	Aβ40 + NSC 69318	-0.89425	0.97967	0.05
Aβ40	Aβ40 + NSC 100873	-0.94498	0.9788	0.05
Aβ40	Aβ40 + NSC 16224	-19.25608	1.79E-07	0.05
Aβ40 + NSC 69318	Aβ40 + NSC 100873	-0.05073	1	0.05
Aβ40 + NSC 69318	Aβ40 + NSC 16224	-18.36183	5.26E-07	0.05
Aβ40 + NSC 100873	Aβ40 + NSC 16224	-18.3111	1.56E-07	0.05

Supplementary Table 2A-B. Analysis of interleukin-6 (IL-6) (A) and tumor necrosis factor (TNF)-α (B) by ELISA after incubation of dTHP-1 cells with $A\beta_{40}$ and the small molecule compounds as shown in Fig. 2. MeanDiff: mean difference between data 1 and data 2, Prob: probability value for significant difference, Alpha: criterion of significance. Significant differences are highlighted in yellow.

A

Data 1	Data 2	MeanDiff	Prob	Alpha
Vehicle	NSC 69318	454	0.99952	0.05
Vehicle	NSC 100873	217.33333	1	0.05
Vehicle	NSC 16224	322.33333	0.99995	0.05
Vehicle	Aβ42	12402	1.46E-07	0.05
Vehicle	Aβ42 + NSC 69318	11534.66667	1.12E-07	0.05
Vehicle	Aβ42 + NSC 100873	12067	1.44E-07	0.05
Vehicle	Aβ42 + NSC 16224	10893.33333	1.31E-07	0.05
NSC 69318	NSC 100873	-236.66667	0.99999	0.05
NSC 69318	NSC 16224	-131.66667	1	0.05
NSC 69318	Aβ42	11948	1.44E-07	0.05
NSC 69318	Aβ42 + NSC 69318	11080.66667	1.22E-07	0.05
NSC 69318	Aβ42 + NSC 100873	11613	1.11E-07	0.05
NSC 69318	Aβ42 + NSC 16224	10439.33333	1.56E-07	0.05
NSC 100873	NSC 16224	105	1	0.05
NSC 100873	Aβ42	12184.66667	1.45E-07	0.05
NSC 100873	Aβ42 + NSC 69318	11317.33333	1.16E-07	0.05
NSC 100873	Aβ42 + NSC 100873	11849.66667	1.44E-07	0.05
NSC 100873	Aβ42 + NSC 16224	10676	1.42E-07	0.05
NSC 16224	Aβ42	12079.66667	1.44E-07	0.05
NSC 16224	Aβ42 + NSC 69318	11212.33333	1.18E-07	0.05
NSC 16224	Aβ42 + NSC 100873	11744.66667	1.09E-07	0.05
NSC 16224	Aβ42 + NSC 16224	10571	1.47E-07	0.05
Aβ42	Aβ42 + NSC 69318	-867.33333	0.97575	0.05
Aβ42	Aβ42 + NSC 100873	-335	0.99994	0.05
Aβ42	Aβ42 + NSC 16224	-1508.66667	0.71661	0.05
Aβ42 + NSC 69318	Aβ42 + NSC 100873	532.33333	0.99866	0.05
Aβ42 + NSC 69318	Aβ42 + NSC 16224	-641.33333	0.99573	0.05
Aβ42 + NSC 100873	Aβ42 + NSC 16224	-1173.66667	0.89247	0.05

B

Data 1	Data 2	MeanDiff	Prob	Alpha
Vehicle	NSC 69318	-0.22667	1	0.05
Vehicle	NSC 100873	-0.20667	1	0.05
Vehicle	NSC 16224	-0.19	1	0.05
Vehicle	Aβ42	37.69667	0	0.05
Vehicle	Aβ42 + NSC 69318	35.54667	3.61E-08	0.05
Vehicle	Aβ42 + NSC 100873	36.46	3.65E-08	0.05
Vehicle	Aβ42 + NSC 16224	32.27667	5.17E-07	0.05
NSC 69318	NSC 100873	0.02	1	0.05
NSC 69318	NSC 16224	0.03667	1	0.05
NSC 69318	Aβ42	37.92333	0	0.05
NSC 69318	Aβ42 + NSC 69318	35.77333	3.62E-08	0.05
NSC 69318	Aβ42 + NSC 100873	36.68667	3.66E-08	0.05
NSC 69318	Aβ42 + NSC 16224	32.50333	1.35E-06	0.05
NSC 100873	NSC 16224	0.01667	1	0.05
NSC 100873	Aβ42	37.90333	0	0.05
NSC 100873	Aβ42 + NSC 69318	35.75333	3.62E-08	0.05
NSC 100873	Aβ42 + NSC 100873	36.66667	3.66E-08	0.05
NSC 100873	Aβ42 + NSC 16224	32.48333	5.22E-07	0.05
NSC 16224	Aβ42	37.88667	0	0.05
NSC 16224	Aβ42 + NSC 69318	35.73667	3.62E-08	0.05
NSC 16224	Aβ42 + NSC 100873	36.65	3.66E-08	0.05
NSC 16224	Aβ42 + NSC 16224	32.46667	5.21E-07	0.05
Aβ42	Aβ42 + NSC 69318	-2.15	0.94687	0.05
Aβ42	Aβ42 + NSC 100873	-1.23667	0.99768	0.05
Aβ42	Aβ42 + NSC 16224	-5.42	0.1679	0.05
Aβ42 + NSC 69318	Aβ42 + NSC 100873	0.91333	0.99967	0.05
Aβ42 + NSC 69318	Aβ42 + NSC 16224	-3.27	0.70016	0.05
Aβ42 + NSC 100873	Aβ42 + NSC 16224	-4.18333	0.42846	0.05

Supplementary Table 3A-B. Analysis of phagocytosis after incubation of dTHP-1 cells with Aβ_{42} and the small molecule compounds as shown in Fig. 3. (A) Average fluorescence intensity and **(B)** percentage of dTHP-1 cells that had taken up Aβ42-488. MeanDiff: mean difference between data 1 and data 2, Prob: probability value for significant difference, Alpha: criterion of significance. Significant differences are highlighted in yellow.
